# *Prototheca zopfii* genotype II induces mitochondrial apoptosis in models of bovine mastitis

**DOI:** 10.1038/s41598-020-57645-z

**Published:** 2020-01-20

**Authors:** Muhammad Shahid, Eduardo R. Cobo, Liben Chen, Paloma A. Cavalcante, Herman W. Barkema, Jian Gao, Siyu Xu, Yang Liu, Cameron G. Knight, John P. Kastelic, Bo Han

**Affiliations:** 1 Department of Clinical Veterinary Medicine, College of Veterinary Medicine, China Agricultural University, Beijing, 100193 P.R. China; 2 Department of Production Animal Health, Faculty of Veterinary Medicine, University of Calgary, Calgary, AB T2N 4N1 Canada; 3 Whiting School of Engineering, Johns Hopkins University, Baltimore, MD 21218 USA; 4 Department of Veterinary Clinical and Diagnositic Sciences, Faculty of Veterinary Medicine, University of Calgary, Calgary, AB T2N 4N1 Canada

**Keywords:** Fungal host response, Fungal immune evasion, Fungal pathogenesis

## Abstract

*Prototheca zopfii* is an alga increasingly isolated from bovine mastitis. Of the two genotypes of *P. zopfii* (genotype I and II (GT-I and -II)), *P. zopfii* GT-II is the genotype associated with acute mastitis and decreased milk production, although its pathogenesis is not well known. The objective was to determine inflammatory and apoptotic roles of *P. zopfii* GT-II in cultured mammary epithelial cells (from cattle and mice) and murine macrophages and using a murine model of mastitis. *Prototheca zopfii* GT-II (but not GT-I) invaded bovine and murine mammary epithelial cells (MECs) and induced apoptosis, as determined by the terminal deoxynucleotidyl transferase mediated deoxyuridine triphosphate nick end labeling assay. This *P. zopfii* GT-II driven apoptosis corresponded to mitochondrial pathways; mitochondrial transmembrane resistance (ΔΨm) was altered and modulation of mitochondrion-mediated apoptosis regulating genes changed (increased transcriptional *Bax*, cytochrome-c and *Apaf-1* and downregulated *Bcl-2*), whereas caspase-9 and -3 expression increased. Apoptotic effects by *P. zopfii* GT-II were more pronounced in macrophages compared to MECs. In a murine mammary infection model, *P. zopfii* GT-II replicated in the mammary gland and caused severe inflammation with infiltration of macrophages and neutrophils and upregulation of pro-inflammatory genes (*TNF-α*, *IL-1β* and *Cxcl-1*) and also apoptosis of epithelial cells. Thus, we concluded *P. zopfii* GT-II is a mastitis-causing pathogen that triggers severe inflammation and also mitochondrial apoptosis.

## Introduction

Bovine mastitis (inflammation of the udder), caused by infection with pathogenic microorganisms and destruction of milk-synthesizing tissues^1^, reduces milk production and quality and is an important financial threat to the dairy industry^2^. *Prototheca zopfii*, a chlorophyllous alga (family *Chlorellaceae*) unable to synthesize chlorophyll and with heterotrophic modes of nutrition^3,4^, is a major cause of mastitis in dairy cows^5,6^. Bovine protothecal mastitis can be clinical or subclinical. In clinical cases, symptoms include fever (up to 40 °C), pain, mammary edema, anorexia and reluctance to move^7^. Subclinical protothecal mastitis is associated with increased number of leukocytes in the udder and milk and can be manifested by slight pain along with loss of appetite^7^. Bovine protothecal mastitis decreases milk production and elevates somatic cell count in milk, especially macrophages, often resulting in culling^7^. Reported bovine *Prototheca zopfii* mastitis occurrence ranges from 7.5 to 16.3%^8,9^; however, these reports are predominantly from outbreaks. Although a large proportion (up to 81%) of dairy herds are infected, this pathogen affects a limited proportion of cows (<10%)^10,11^. Cows are often infected intramammarily with *P. zopfii* following teat trauma during mechanical milking^12^ and contamination of the teat orifice with damp organic material^7,13^. Single *Prototheca zopfii* endospores or sporangiospores contact mammary gland epithelial cells, which are first responders, sensing their presence and initiating an inflammatory immune response. After breaching epithelial defenses, *Prototheca zopfii* may also invade macrophages of the mammary gland alveolar lumen and interstitium^14^, making *Prototheca zopfii* less accessible to antibiotics and diagnostic methods^15^.

Two genotypes of *Prototheca zopfii*, genotype I (GT-I) and genotype II (GT-II) have been isolated from bovine milk and identified^16^. Genotype I is predominantly isolated from environmental samples, whereas GT-II is isolated from milk samples and has been reported as the causative pathogen of bovine mastitis^17–19^. In the latest study, the two types were named *P. ciferri* and *P. bovis*, separately^20^. *Prototheca zopfii* GT-II induced oxidative stress and apoptotic death in cultured bovine mammary epithelial cells (bMECs), *Prototheca zopfii* GT-II is more pathogenic than *P. zopfii* GT-I, commonly isolated as an enviromental apathogenic microbe^21,22^. Moreover, another study reported that mammary gland infected with *P. zopfii* GT-I had no clinical signs^14^, but pathogenesis of protothecal mastitis due to *P. zopfii* GT-II remains elusive. Thus, we aim to determine inflammatory and apoptotic roles of *Prototheca zopfii* GT-II in cultured mammary epithelial cells (from cattle and mice) and murine macrophages and using a murine model of mastitis.

## Materials and Methods

### Statement of ethics

The current study was conducted in accordance with ethical guidelines and regulations regarding laboratory animal care and use, as described in the “Guide to the Care and Use of Experimental Animals” from the Canadian Council on Animal Care (https://www.ccac.ca/Documents/Standards/Guidelines/Experimental_Animals_Vol1.pdf). Animal use was reviewed and approved by the Animal Care Committee of the University of Calgary, Calgary, AB, Canada (protocol number AC16–0061).

### *Prototheca zopfii* culture

*Prototheca zopfii* GT-II isolates were collected from milk samples of dairy cows with clinical mastitis, whereas *P. zopfii* GT-I isolates were predominantly cultured from environmental samples in China, and cultured and stored at College of Veterinary Medicine, China Agricultural University, Beijing, China^23^. *P. zopfii* GT-I and II were isolated from a total of 163 *P. zopfii* isolates collected from mastitic milk and environmental samples^18^. In this study, *P. zopfii* GT-II was only isolated from mastitic milk, whereas GT-I was recovered from environmental samples (feed, feces, water and teat cups). Both genotypes were characterized by their cellular fatty acid pattern and 18 S rDNA sequences. *P. zopfii* GT-II had increased amounts of eicosadienoic acid (C20: 2) compared to GT-I. Whereas both *P. zopfii* GT-I and II had high sequence similarity (99.4%), GT-II (AY940456) differed in some nucleotides from GT-I (AY973040)^16^. All bovine mastitis milk *P. zopfii* strains were further identified by genotype-specific PCR and restriction fragment length polymorphism analysis^16,24^. In our previous study, the 450 bp fragment internal amplification control was detected using *Proto18–4f* (GACATGGCGAGGATTGACAGA) and *Proto18–4r* (AGCACACCCAATCGGTAGGA) sequences. The GT-I strain was identified by *Proto18–4f* (GACATGGCGAGGATTGACAGA) and *PZGT-1/r* (GCCAAGGCCCCCCGAAG) primers. GT-II specific amplicon (165 bp) was detected with primers *Proto18–4f* (GACATGGCGAGGATTGACAGA) and *PZGT-II/r* (GTCGGCGGGGCAAAAGC)^18^. The *P. zopfii* genotype was further confirmed by restriction fragment length polymorphism (RFLP) analysis targeting the *cytb* gene fragment (599–668 bp)^5^. For this, a PCR mix (25 µL) containing cytb-F1 (5′ GyGTwGAACAyATTATGAGAG-3′) and cytb-R2 (5′-wACCCATAArAArTACCATTCwGG-3′) primers (10 μM each primer), DNA template (1 µL), and 2x EasyTaq PCR supermix (TransGen Biotech, AS111–11; 12.5 µL) was amplified under specific conditions (2 min at 95 °C, followed by 35 cycles of 30 sec at 95 °C, 30 sec at 50 °C, and 30 sec at 72 °C, with final extension of 5 min at 72 °C). The PCR products depicted a 644 base pair (bp) product compatible with *P. zopfii* as visualized by agarose gel electrophoresis (1%, wt/vol) and stained with ethidium bromide. The amplified *cytb* gene products (644-bp) were digested by RsaI and TaiI digesting enzymes (FastDigest Enzymes, Thermo Fisher Scientific). The total mixture (30 µL) containing 10x restriction enzyme buffer (3 µL), PCR product (10 µL), enzymes (1.5 µL each) and PCR water (16.5 µL) was digested by RsaI (5 min at 37 °C) followed by TaiI (5 min at 65 °C). The restriction products visualized on 4% agarose gels, stained with ethidium bromide, and exposed to UV light showed DNA fragments of 200 and 450 bp after RSaI/TaiI digestion, compatible with *P. zopfii* GT -II (Supplementary Fig. 1). Taken together, we confirmed a *P. zopfii* II genotype in the isolate clinically recovered from a case of mastitis in cows. Prior to each experiment, fresh *P. zopfii* GT-I and -II were cultured on Sabouraud dextrose agar (SDA; Sigma, Shanghai, China) for up to 48 h at 37 °C and single colonies incubated in Sabouraud dextrose broth (SDB; Sigma) at same conditions for up to 72 h^23^.

### Mouse protothecal mastitis model

C57BL/6 lactating female mice (6–8 wk old; 10–14 d after parturition) were housed in specific pathogen-free facilities at the University of Calgary with *ad libitum* access to food and water. Mice were inoculated intramammarily with either *P. zopfii* GT-II (50 µL containing 1 × 10^5^ CFU/mL) or an equal volume of phosphate buffered saline (PBS) (control) in the left fourth and right fourth (L4 and R4) mammary glands. Mice were euthanized 4 d post inoculation (dpi) to collect mammary tissue samples. Tissues were mixed into TRIzol (Invitrogen, Carlsbad, CA, USA) and later homogenized for quantitative PCR (qPCR) or fixed in 10% formalin solution, embedded in paraffin wax, sectioned with a microtome (5 µm) and stained with hematoxylin and eosin (H&E; Sigma, USA) for histological examination^25^ and with Periodic Acid-Schiff (PAS; Sigma, USA) and Grocott-Gomori’s methenamine silver stain (GMS) as a screen for fungal organisms.

### Identification of macrophages and neutrophils in murine mammary gland

Fixed murine mammary gland tissue sections were deparaffinized, dehydrated and permeabilized with PBS/Triton X-100 (0.25%, v/v) (PBS-T) buffer containing 1% donkey serum (Cat # 017–000–121) at room temperature for 10 min. Slides were blocked with PBS-T containing 10% (v/v) donkey serum and 1% (v/v) bovine serum albumin (BSA) (Sigma, USA) for 120 min at room temperature. After washing with PBS, sections were incubated with primary antibodies against murine F4/80 (macrophages) (Cat # 4316835, BD Pharmingen™, US) and Ly-6G (neutrophils) antigens (Cat# 127609, Biolegend, US) (1:1,000 in PBS-T plus 1% BSA) for 16 h at 4 °C. Following washing with PBS-T, slides were incubated with secondary antibodies (488-conjugated Affinipure Goat anti-Rat IgG, Cat# 135205, Jackson Immune Research, UK) (1:1,000 in PBS-T plus 1% BSA) at room temperature for 60 min and washed again with PBS-T and then incubated with DAPI (4′, 6-diamidino-2- phenylindole) (Invitrogen) at room temperature for 20 min. Slides were examined with an immunofluorescence microscope (ZEISS Axio Imager M2, Carl Zeiss AG, Jena, Thuringia, Germany).

### Epithelial cell and macrophage culture

A bMEC line isolated from a cow (MAC-T) (Shanghai Jingma Biological Technology Co., Ltd. China), murine macrophages derived from mouse BALB/c monocytes (J.774, provided by Dr. Eduardo R. Cobo, University of Calgary) and a murine mammary epithelial cells line (mMECs; HC11, provided by Dr. Eduardo R. Cobo, University of Calgary) were used. The bMECs and murine macrophages were cultured in HyClone ^TM^ DMEM/F12 medium (Thermo Fisher Scientific, South Logan, NH, USA) along with 10% fetal bovine serum (FBS; Thermo Fisher Scientific), penicillin (100 U/mL; Thermo Fisher Scientific) and streptomycin (100 U/mL; Thermo Fisher Scientific) in cell culture plates (Corning Inc., Corning, NY, USA). The mMECs were cultured in RPMI (Thermo Fisher Scientific) medium along with 10% fetal bovine serum (FBS; Thermo Fisher Scientific), penicillin (100 U/mL; HyClone^®^, USA) and streptomycin (100 U/mL; Thermo Fisher Scientific). For experimental challenges, bMECs and macrophages (bovine and murine) were challenged with *P. zopfii* GT- I and GT-II suspended in DMEM/F12 to 5 × 10^5^ and 1 × 10^5^ CFU/mL, respectively, for up to 24 h at 37 °C with 5% CO_2_.

### *P. zopfii* cell internalization assay

Murine macrophages and bMECs were infected with *P. zopfii* for up to 8 h, washed with PBS (pH 7.4) and incubated for 2 h with gentamycin (200 μg/mL) to eliminate extracellular *P. zopfii*. Cells were washed with PBS to eliminate non-adherent bacteria and then lysed with 0.5% Triton X-100 (v/v) to determine CFU by 10-fold serial dilution^26^. Further confirmation of phagocytic activity of macrophages was done by actin inhibition (cytochalasin D; C8273, Sigma, USA; 1 h) before inoculation.

### Transmission electron microscopy (TEM)

Bovine MECs infected with *P. zopfii* GT-I and -II were washed with PBS (pH 7.2), fixed with 2% glutaraldehyde and 1% paraformaldehyde (pH 7.2; Sinopharm Chemical Reagent Co., Shanghai, China) and processed for TEM^22^.

### Mitochondrial damage assay

After infection with *P. zopfii*, GT-I and -II, bovine MECs were collected to assess changes in mitochondrial membrane potential (ΔΨm), as determined by presence of JC-1 (Cat# M8650, Solarbio, Beijing, China) using flow cytometry and immunofluorescence microscopy. JC-1 is a dual-emission potential-sensitive probe that forms red-fluorescent aggregates in healthy mitochondria, but becomes a green-fluorescent monomer after membrane potential collapses.

### Transcriptional gene expression of inflammatory and apoptotic genes

Total RNA was extracted from bMECs, mMECs and murine macrophages with TRIzol reagent (Invitrogen) and converted to cDNA (RevertAid First Strand cDNA synthesis kit, Thermo Scientific). Quality of resulting RNA and cDNA were evaluated by the absorbance ratio (A260/A280 ratio) (NanoVue Spectrophotometer, GE Healthcare Bio-Sciences, Little Chalfont, Buckinghamshire, UK)^27^, which was corrected to be ∼1.8–2.0 for an individual sample. Amplification of mRNA genes for *TNF-α*, *IL-1β*, *IL-8/Cxcl-1*, *Bcl-2*, *Bax*, *Apaf-1*, cytochrome-c, caspase-9 and caspase-3 was done using a CFX-96 real-time PCR system (BioRad, Hercules, CA, USA). The reaction mixture for each sample carried 2 µL of cDNA, 1X SsoAdvanced Universal SYBR Green Supermix (BioRad) and 0.5 μM of each specific primer, in a 10 μL final volume. Relative primers for bovine and murine genes are shown (Tables 1 and 2, respectively). Reaction mixtures were incubated at 95 °C for 5 min, followed by denaturation for 5 s at 95 °C and combined annealing/extension for 10 s at 60 °C (total of 40 cycles). All treatments were examined in duplicate in three independent experiments. Values of target mRNA were corrected relative to the normalizer *GAPDH*. Data were assessed using the 2−ΔΔCT method^27^ and results presented as mean fold change of target mRNA levels in infected groups versus an uninfected control group^27^.

**Table 1:** Primer sequences of qPCR for bovine genes.

Gene	Primer sequence (5′−3′)	
*TNF-α*	5′ACGGGCTTTACCTCATCTACTC	3′GCTCTTGATGGCAGACAGG
*IL-1β*	5′AGGTGGTGTCGGTCATCGT	3′GCTCTCTGTCCTGGAGTTTGC
*IL-8*	5′ACACATTCCACACCTTTCCA	3′GGTTTAGGCAGACCTCGTTT
*Apaf-1*	5′ACCTTGTTGGCGACTG	3′TTCTACTGAAATCGGAGC
Caspase-9	5′GCAGTGGACGCTGGTTCT	3′TTGCTTGGCAGTCAGGTC
Caspase-3	5′GAGCCTGTGAGCGTGCTTTT	3′TGGTGCTGAGGATGACATGG
*Bcl-2*	5′ATGTGTGTGGAGAGCGTCAA	3′GGGCCATACAGCTCCACAAA
*Bax*	5′GCGCATCGGAGATGAATTGG	3′AGATGGTCACTGTCCAACCAC
*GAPDH*	5′CATTGACCTTCACTACATGGT	3′ACCCTTCAAGTGAGCCCCAG

**Table 2:** Primer sequences of qPCR for murine genes.

Gene	Primer sequence (5′−3′)	
*TNF-α*	Cat # PPM03113G-200	
*IL-1β*	Cat # PPM03109F-200	
*Cxcl-1*	Cat # PPM03058C-200	
*Apaf-1*	5′TCCAGCGGCAAGGACACAGACG	3′CAACCGCGTGCAAAGATTCTGCA
Cytochrome-c	5′GGCTGCTGGATTCTCTTACAC	3′GTCTGCCCTTTCTCCCTTCT
Caspase-9	5′CTGAGCCAGATGCTGTCCCATA	3′CCAAGGTCTCGATGTACCAGGAA
Caspase-3	5′ACTGGAAAGCCGAAACTCTTC	3′CATACAGGAAGTCAGCCTCCA
*Bcl-2*	5′ATGTGTGTGGAGAGCGTCAAC	3′CAGCCAGGAGAAATCAAACAG
*Bax*	5′GAGACACCTGAGCTGACCTTG	3′GAAGTTGCCATCAGCAAACAT
*GAPDH*	5′AAATGGTGAAGGTCGGTGTG	3′TGAAGGGGTCGTTGATGG

### TUNEL apoptosis staining

Apoptosis of bMECs, mMECs, murine macrophages and mouse mammary gland after *P. zopfii* GT-II inoculation was assessed by *in situ* TUNEL staining (S7165 ApopTaq apoptosis detection kit, MilliporeSigma, Haverhill, MA, USA). Apoptotic indices were calculated as positive stained apoptotic cells per field, using five fields per sample at 400 × magnification.

### Protein determination of apoptotic cytochrome-c, caspase-9, and caspase-3

Proteins from bMECs or homogenized murine mammary tissue were size-separated by SDS-PAGE and transferred to Immobilon-P polyvinylidene difluoride (PVDF) membrane (0.45 µm) (Millipore Sigma, Gillingham, Dorset, UK). Membrane was blocked with 5% skim milk in TBS-T (150 mM NaCl, 10 mM Tris base, 0.05% Tween 20, pH 7.4) at room temperature for 120 min and then incubated overnight at 4 °C with primary antibodies for caspase-9 (Cat # ab69514, Abcam USA), caspase-3 (Cat # ab90437, Abcam USA), cytochrome-c (Cat # ab110325, Abcam USA) and housekeeping β-tubulin (Cell Signaling Technology, Danvers, MA, USA). The membrane was rinsed with TBS-T and incubated with HRP-labeled secondary goat anti-rabbit IgG (ZRA03, Biotech, China) or goat anti-mouse IgG (ZM03, Biotech, China) at 37 °C for 60 min. Signals were detected using enhanced chemiluminescence (Cat # PE0010, Solarbio Life Sciences, Beijing, China).

### Protein detection by ELISA

Secreted *Cxcl-1* and *TNF-α* proteins in infected and control mice were quantified by ELISAs (DuoSet ELISA # DY453–05 and # DY410–05, R&D Systems, Minneapolis, MN, USA).

### Statistical analyses

Data were analyzed in triplicate for reproducibility and were expressed as mean ± standard deviation (SD). Data from infected and uninfected groups were analyzed using a paired Student’s *t-*test with a 95% confidence interval. Data were further analyzed by ANOVA and *post hoc* tests using SPSS 20.0 (International Business Machines Corporation, Armonk, NY, USA). For all analyses, *P* < 0.05 was considered significant.

## Results

### *P. zopfii* GT-II induced mastitis and apoptosis in a mouse model

**Figure 1:**
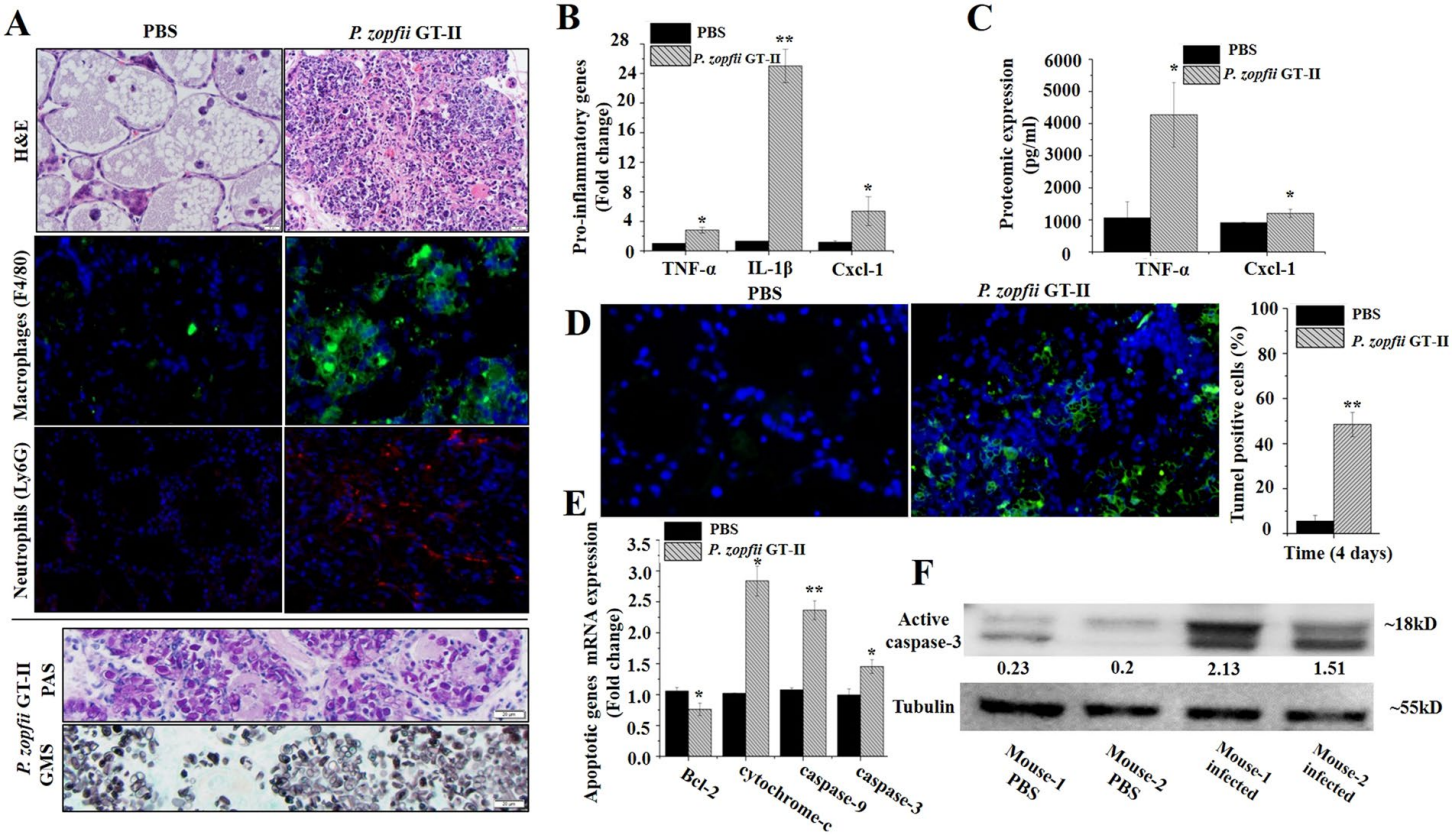
*Prototheca zopfii* genotype II-induced microscopic changes in the mammary gland of mice. (**A**) H&E, PAS and GMS staining and immune detection of macrophages (F4/80) and neutrophils (Ly6 G) in PBS control and *P. zopfii*-infected mammary tissues at 4 d post infection. Note infiltration of macrophages and neutrophils and presence of innumerable *P. zopfii* GT-II as detected by PAS and GMS staining. Bar = 20 µm. (**B**) Transcriptomic expression of genes of pro-inflammatory *TNF- α, IL-1β* and *Cxcl-1* after infection with *P. zopfii* GT-II. (**C**) ELISA titers of *TNF-α* and *Cxcl-1* in mammary tissue. (**D**) Quantitative detection of apoptotic cells in PBS control and *P. zopfii* GT-II infected mammary tissue in mouse mammary gland (green signal indicates TUNEL apoptotic cells). (**E**) Transcriptomic expression of *Bcl-2*, cytochrome-c, caspase-9, and caspase-3 in mouse mammary tissues infected with *P. zopfii* GT-II. (**F**) Proteomic expression of activated caspase-3 in mammary gland 4 d after *P. zopfii* GT-II infection, tubulin and activated caspase-3 run on different gels at same time and cropped according to respective size. *P < 0.05, **P < 0.01.

To investigate causative effects of *P. zopfii* GT-II in protothecal mastitis, lactating mice were intramammarily challenged with *P. zopfii* GT-II isolated from a bovine clinical mastitis case. Round to oval sporangia with regular internal divisions compatible with *P. zopfii* were observed in the mammary gland of lactating mice at 4 dpi, as detected by PAS and GMS staining (Fig. 1A). *Prototheca zopfii* GT-II replicated in the murine mammary gland as it was recovered by culture in greater amounts at 4 dpi compared to the initial inoculum (mean 3.4 × 10^7^ CFU/g tissue).

*Prototheca zopfii* GT-II induced acute mastitis with infiltration of leukocytes throughout the parenchyma and within lumina of alveoli. *Prototheca zopfii* GT-II were present both free within alveolar lumina and throughout the interstitium of the mammary tissue (Fig. 1A). Using immune detection, macrophages were demonstrated in the mammary interstitium and neutrophils diffusely distributed in *P. zopfii* GT-II-infected mice (Fig. 1A). The presence of *P. zopfii* GT-II upregulated gene activity and protein production of pro-inflammatory *TNF-α, IL-1β* and *Cxcl-1* in mammary tissue at 4 dpi (Fig. 1B,C).

Next, we determined whether intramammary infection with *P. zopfii* GT-II involved apoptosis and oxidative stress, as described in cultured bovine mammary epithelial cells (bMECs)^20,21^. Apoptotic cells were quantified at 4 dpi with *P. zopfii* GT-II (Fig. 1D). Transcriptomic analysis demonstrated that mRNA expression of caspase-9 and caspase-3 genes regulating mitochondrion-mediated apoptosis was higher in *P. zopfii* GT-II infected mice (Fig. 1E) with cleavage of caspase-3 protein (Fig. 1F). Expression of *Bax* gene increased in mammary tissue after *P. zopfii* GT-II inoculation (Supplementary Fig. 2A), whereas expression of *Bcl-2* decreased (Fig. 1E). Expression of cytochrome-c released into the cytosol to trigger apoptosis (Fig. 1E) and *Apaf-1* also increased in *P. zopfii* GT-II inoculated mice (Supplementary Fig. 2B).

### *P. zopfii* GT-II-driven apoptosis occurred in both mammary epithelial cells and macrophages

**Figure 2:**
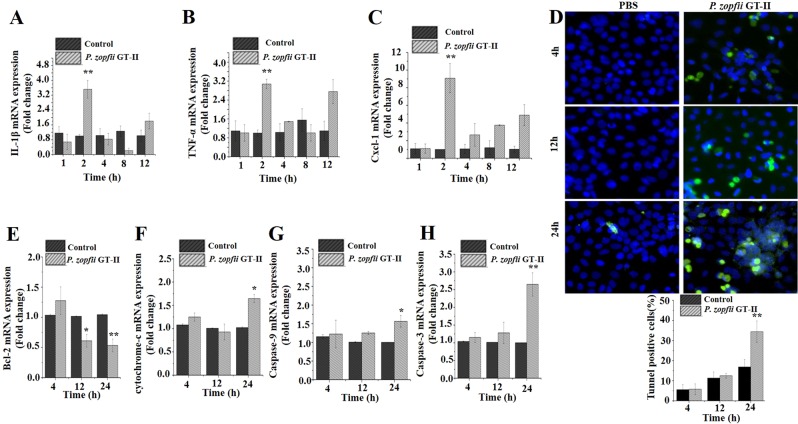
Murine MECs infected with *Prototheca zopfii genotype II* (GT-II). (**A–C**) Expression of mRNA level of *TNF-α, IL-1β*, and *Cxcl-1* in murine MECs was quantified after infection of *P. zopfii* GT-II infection. (**D**) Representative picture of TUNEL assay, plus quantitative analysis of TUNEL-positive apoptotic cells (20×). (**E–H**) mRNA expression of *Bcl-2*, caspase-9 and caspase-3, respectively, was quantified by qPCR and expressed as fold change relative to uninfected cells. Data are mean ± SD of three independent experiments. *P < 0.05, **P < 0.01.

Since mastitis is a process involving epithelial cells and leukocytes, we investigated contributions of single-cell components in pathogenesis of *P. zopfii* GT-II mastitis and apoptotic responses. We used a murine MEC (HC11) with ability to produce milk proteins (beta-casein) in response to prolactin^28^. Infection with *P. zopfii* GT-II in MEC induced early *IL-1β, TNF-α* and *Cxcl-1* gene expression (after 2 hpi) (Fig. 2A–C). Apoptotic cells appeared later (24 hpi; Fig. 2D) with an increased transcriptional expression of hallmark apoptotic genes, *Bax*, *Apaf-1* (Supplementary Fig. 2C,D), cytochrome-c, caspase-9 and −3 genes (Fig. 2E–G). Expression of *Bcl-2* was reduced (Fig. 2H).

**Figure 3:**
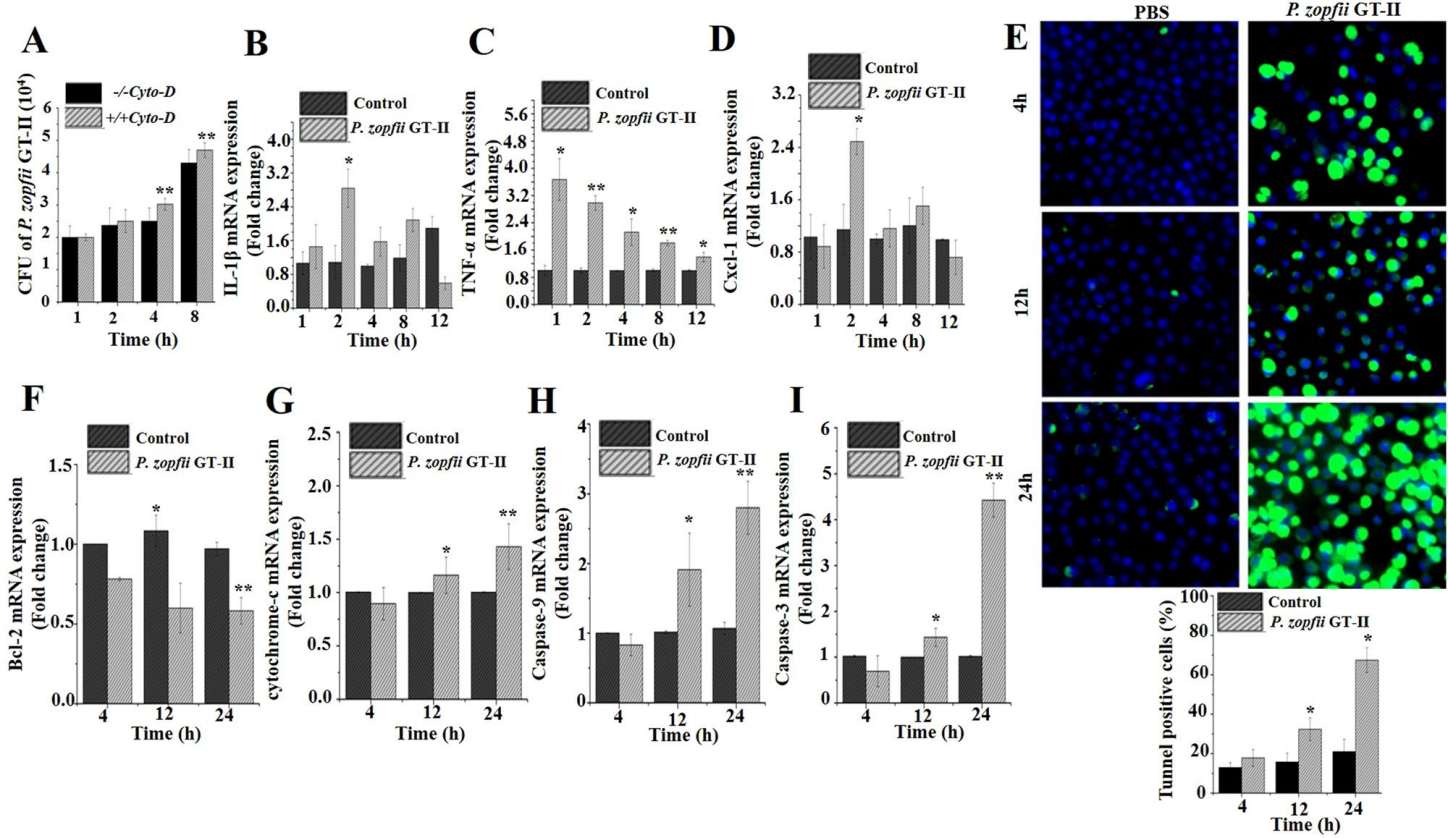
Murine macrophages infected with *P. zopfii* GT-II. (**A**) Internalization of *P. zopfii* GT-II in mouse macrophages in time-dependent manner, with and without cytochalasin D. (**B–D**) Level of cytokines (*TNF-α, IL-1β* and *Cxcl-1*) in murine macrophages. (**E**) TUNEL assay of mouse macrophages, quantitative analysis of apoptotic positive cells TUNEL positive apoptotic cells (20×). (**F–I**) Transcriptomic expression of *Bcl-2*, cytochrome-c, caspase-9, and caspase-3 at 4, 12 and 24 h after infection with *P. zopfii* GT-II in mouse macrophages on qPCR analysis and expressed as fold change relative to uninfected cells. Data are mean ± SD of three independent experiments. *P < 0.05, **P < 0.01.

To examine the role of macrophages, key in chronic mastitis^29^, murine macrophages (J774) with phagocytic characteristics were challenged with *P. zopfii* GT-II. *Prototheca zopfii* GT-II internalized inside macrophages in a time-dependent fashion (up to 8 hpi; Fig. 3A). This internalization seemed to be an active microbe process (*P. zopfii* dependent) rather than a phagocytic event, as actin inhibition in macrophages (by cytochalasin D) did not prevent *P. zopfii* GT-II internalization (Fig. 3A). Infection of *P. zopfii* GT-II in macrophages upregulated mRNA expression of *IL-1β, TNF-α* and *Cxcl-1* (2 h; Fig. 3B–D). In contrast, *TNF-α* expression decreased over time (Fig. 3C). *Prototheca zopfii* GT-II induced apoptosis in macrophages as detected by TUNEL assay, with more cell death at 12 and 24 hpi (Fig. 3E) and upregulated expression of *Bax, Apaf-1* (Supplementary Fig. 2E,F), cytochrome-c, caspase-9, and caspase-3 genes, whereas *Bcl-2* expression decreased in a time-dependent manner (Fig. 3F–I).

### *P. zopfii* GT-II induced apoptosis in bovine mammary epithelial cells

**Figure 4:**
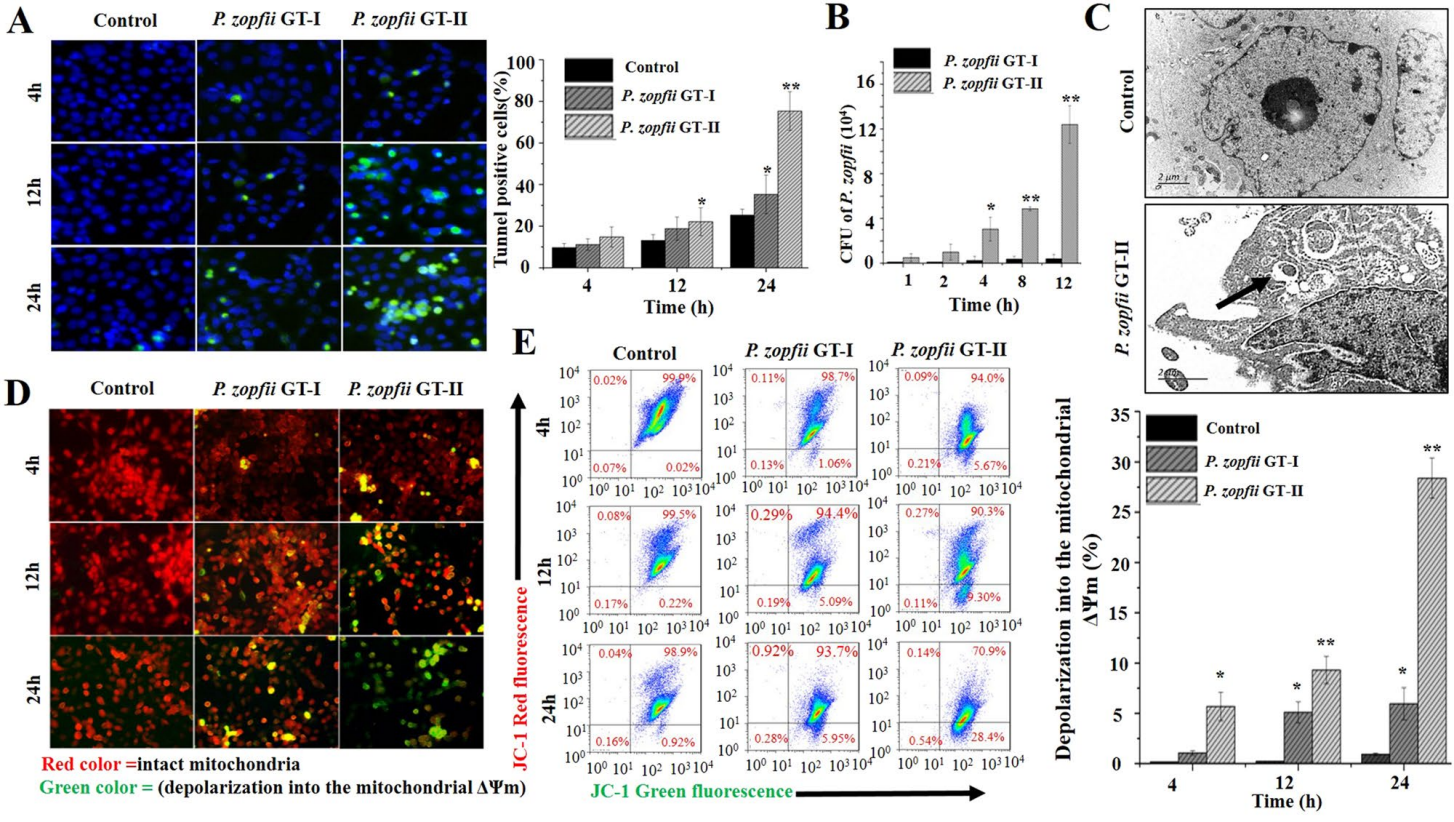
Bovine mammary epithelial cells (bMECs) *Prototheca zopfii genotype* (GT)-I and -II *in vitro* infection model. (**A**) Quantitative detection of apoptotic cells in *P. zopfii* GT-I and -II infected bovine mammary epithelial cells (green signal indicates TUNEL apoptotic cells). (**B**) Internalization of *P. zopfii* GT-II in bMECs was increased in a time-dependent fashion as compared to *P. zopfii* GT-I infection in bMECs. **(C)** Intracellular localization of *P. zopfii* GT-II in bMECs on transmission electron microscopy (black arrow). (**D**) Mitochondrial transmembrane potential (ΔΨm) assay of bMECs infected with *P. zopfii* using JC-1 staining (the compound 5,5′,6,6′-tetrachloro-1,1′,3,3′-tetraethyl-imidacarbocyanine iodide (JC-1), which selectively enter into the mitochondria, formed monomers (green color), indicative of depolarization into the mitochondrial membrane potential (ΔΨm) (remain as multimer J-aggregates (red color) in intact mitochondria). ΔΨm was analyzed by immunofluoresnce microscopy. (**E**) ΔΨm was evaluated by flow cytometry and percentile values of ΔΨm induced by *P. zopfii* in bMECs. *P < 0.05, **P < 0.01.

To verify apoptotic effects of *P. zopfii* in the target animal species (cattle), prototype bovine MECs with morphological and functional characteristics of normal mammary epithelial cells were challenged with *P. zopfii* GT-II and GT-I common commensals in farm environments (e.g., animal bedding, soil)^3^. *Prototheca zopfii* GT-I did not induce any apoptotic effects, but *P. zopfii* GT-II caused TUNEL-mediated apoptosis in a time-dependent manner (Fig. 4A). This occurred rapidly, as *P. zopfii* GT-II were internalized by bMECs in the first 4 hpi, as confirmed by culture (Fig. 4B) and TEM (Fig. 4C). Apoptotic effects induced by *P. zopfii* GT-II were likely of mitochondrial origin, as mitochondrial transmembrane depolarization was detected by immunofluorescence and flow cytometry (12–24 hpi; Fig. 4D–E).

**Figure 5:**
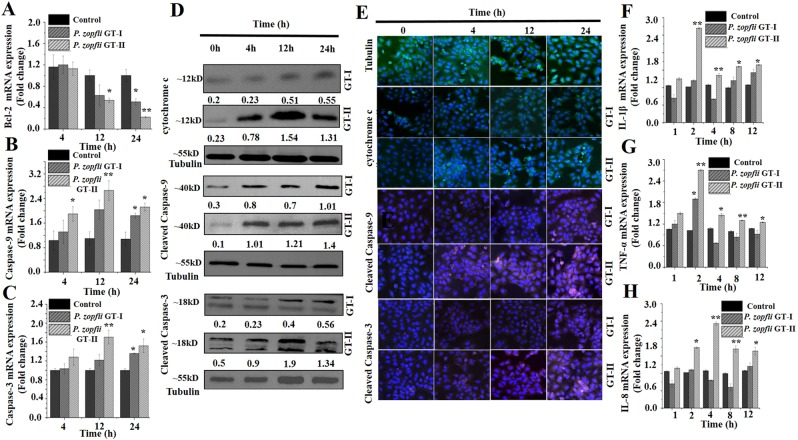
*In vitro* infection of bovine mammary epithelial cells (bMECs) with *Prototheca zopfii* genotype (GT)-I and -II. (**A–C**) Transcriptomic analysis of *Bcl-2*, caspase-9 and caspase-3, respectively. (**D**,**E**) Western blot and confocal laser scanning microscopic analysis of cytochrome-c, caspase-9 and caspase-3 in bMECs, in western blot each samples run on two gels, for control and respective target antigen and cropped according to size of antibodies. (**F,H**) mRNA expression of pro-inflammatory cytokines (*TNF-α, IL-1β* and *IL-8*) quantified by qPCR in bMECs after infection of *Prototheca zopfii* genotype -I and -II infection. *P < 0.05, **P < 0.01.

Transcriptional expression of genes regulating mitochondrion-mediated apoptosis, including increased *Bax* and *Apaf-1* (Supplementary Fig. 2G,H) and decreased *Bcl-2*, were detected in bovine MECs inoculated with *P. zopfii* GT-II (Fig. 5A). Expression of caspase-9 mRNA at early points (4 hpi) followed by caspase-3 mRNA later (24 hpi), increased after *P. zopfii* GT-II infection (Fig. 5B,C). Likewise, cytochrome-c and cleaved caspase-9 and-3 were over time increasingly immune blotted (Fig. 5D) and immunolocalized (Fig. 5E) in bMECs infected with *P. zopfii* GT-II. Apart from decreased *Bcl-2* expression after 24 hpi, no effect of *P. zopfii* GT-I on apoptotic genes in bMECs was observed (Fig. 5A).

Infection of *P. zopfii* GT-II induced pro-inflammatory responses in bMECs as demonstrated by upregulated mRNA expression of *IL-1β, TNF-α* and *IL-8* (after 2 hpi; Fig. 5F,H). However, GT-I did not modify any inflammatory cytokine response in bMECs and demonstrated the apthogenic nature of this *Prototheca*. Taken together, *P. zopfii* GT-II was demonstrated to cause udder disease by provoking apoptosis and inducing inflammatory cytokine expression in mammary epithelium.

## Discussion

Previously, pathogenesis of protothecal mastitis and virulence of *P. zopfii* GT-II isolated from bovine milk were uncertain. In this study, we used a *Prototheca spp*. identified as *P. zopfii* GT-II following a taxomonic approach commonly accepted for Prototheca^16^ and a cytb-based genotyping used for unambiguous *Prototheca spp*. identification^5^ based on the protothecal phylogeny^20^ and we described the pathogenic role of *P. zopfii* GT-II when initiating acute mastitis and mitochondrion-mediated apoptosis using a murine mastitis model and cultured mammary epithelial cells and macrophages. Our study demonstrated that *P. zopfii* GT-II invaded mammary parenchyma and caused acute mastitis, with severe infiltration of macrophages and neutrophils and marked epithelial damage. A destructive role of *P. zopfii* GT-II has been reported in the udder interstitium of cows and mammary acini of mice experimentally infected with *P. zopfii* GT-II^30,31^.

Mammary epithelial cells are essential in microbial infection for sensing pathogens and producing an array of inflammatory cytokines^32^. Pro-inflammatory cytokines, including *TNF-α*, *IL-1β, IL-6* and *IL-8*, have direct cytopathic effects leading to tissue damage^33^. Additionally, *IL-1β* and *TNF-α* can induce cell apoptosis^34^. *Prototheca zopfii* GT-II infection triggered expression of *IL-1β, Cxcl-1/IL-8*, and *TNF-α* in murine macrophages and bMECs. Thus, *P. zopfii* GT-II provoked apoptosis of bMECs by inducing *IL-1β* and *TNF-α* release in macrophages and mammary epithelial cells. *Prototheca zopfii* GT-II was more pathogenic than *P. zopfii* GT-I, commonly isolated as an enviromental apathogenic microbe. *Prototheca zopfii* GT-II induced more *IL-8* mRNA in bMECs compared to GT-I-inoculated or uninfected cells. Increased levels of *IL-8* mRNA in murine MECs and bovine MECs induced by *P. zopfii* GT-II demonstrated that mammary epithelial cells are an important source of *IL-8* and that this chemokine is key during protothecal mastitis, perhaps by recruiting leukocytes, as demonstrated by its chemoattractant role in *Staphylococcus aureus* infection in bMECs^35,36^.

**Figure 6:**
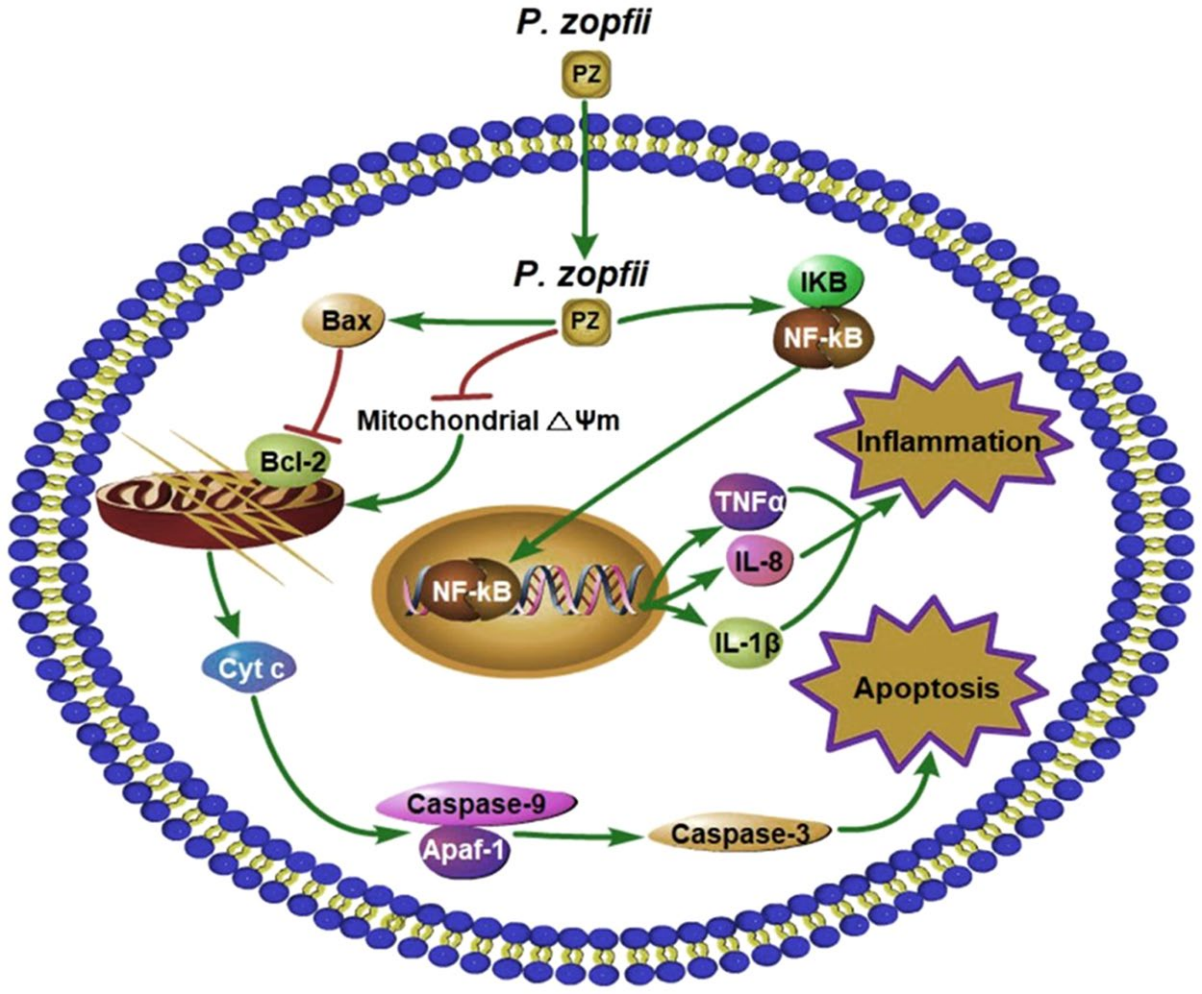
Schematic presentation of mitochondrial-caspase induced apoptosis and inflammation. Depolarization of mitochondrial transmembrane (ΔΨm) causes the release of cytochrome-c, which may initiate caspase cascade. Cytochrome-c bonds with apoptotic protease-activating factor 1 (*Apaf-1*) and activates caspase-9, this cleaves and activates caspase-3, which triggers apoptosis. NF-κB subunit 65 transiting into the nucleus wherein it regulates transcription of pro-inflammatory genes, e.g. *IL-1β* and *TNF-α*.

Whereas *P. zopfii* has been reported to induce apoptosis in cultured bMECs^21,22^, we demonstrated the pro-apoptotic role of *P. zopfii* GT-II in a murine mastitis model. The pro-apoptotic character of *P. zopfii* GT-II was demonstrated by increased numbers of TUNEL-positive cells in *P. zopfii* GT-II-infected mice, along with reduced *Bcl-2* levels and elevated transcriptomic levels of *Bax*, *Apaf-1*, caspase-3, and caspase-9. These all indicated apoptosis via the intrinsic pathway, with functional alterations in mitochondria in mammary epithelial cells infected with *P. zopfii* GT-II. Moreover, *P. zopfii* GT-II induced ROS generation^21^ which triggers mitochondrial *Bax*, a proapoptotic element of the *Bcl-2* family proteins^37^. *Prototheca zopfii* GT-II invaded bMECs and murine macrophages, and indeed, apoptotic effects were promoted by microbial internalization, but independent of phagocytosis. *Prototheca zopfii* GT-II had higher penetration capabilities in bMECs than *P. zopfii* GT-I. We propose that mitochondrial damage due to *P. zopfii* GT-II invasion released protein cytochrome-c from intermembrane spaces into cytosol, which bonded with *Apaf-1* to initiate apoptosome formation and activation of caspase-9 and caspase-3^38–40^. Such *P. zopfii*-driven apoptosis was not restricted to mammary epithelial cells but also applied to leukocytes, including murine macrophages. Whereas *P. zopfii* GT-II was a pathogenic type of *Prototheca* causing mastitis, studies with other *Prototheca* strains may elucidate the complexity of these algae and their interactions with host and enviroment. A hypothetical schematic illustration of mitochondrial caspase-induced apoptotic pathway and NF-κB subunit 65 transiting into the nucleus in protothecal mastitis (Fig. 6) was consistent with reports in bMECs, wherein *P. zopfii* GT-II regulated transcription of pro-inflammatory genes like *IL-1β* and *TNF-α*^35^. In conclusion, pathomorphological alteration caused by *P. zopfii* GT-II highlighted this gentoype as a mastitis pathogen capable of penetrating into mammary epithelial cells to induce inflammation and cell death, via mitochondrial-dependent apoptosis.

## Supplementary information

Supplementary Figure 1.

Supplementary Figure 2.
