# Supplementary material for: Prototheca zopfii genotype II induces mitochondrial apoptosis in models of bovine mastitis
Source: Sci Rep. 2020 Jan 20;10:698. doi: 10.1038/s41598-020-57645-z (PMC6971270; doi:10.1038/s41598-020-57645-z)

Original articles for submission to *Scientific Reports-*

Prototheca zopfii genotype II induces mitochondrial apoptosis in models of bovine mastitis

Muhammad Shahid[1], Eduardo R. Cobo[2], Liben Chen[3], Paloma A. Cavalcante[2], Herman W. Barkema[2], Jian Gao[1], Siyu Xu[1], Yang Liu[1], Cameron G. Knight[4], John P. Kastelic[2], Bo Han[1]*

[1]Department of Clinical Veterinary Medicine, College of Veterinary Medicine, China Agricultural University, Beijing 100193, P.R. China

[2]Department of Production Animal Health, Faculty of Veterinary Medicine, University of Calgary, Calgary, AB, Canada, T2N 4N1

[3]Whiting School of Engineering, Johns Hopkins University, Baltimore, MD 21218, USA

[4]Department of Veterinary Clinical and Diagnositic Sciences, Faculty of Veterinary Medicine, University of Calgary, Calgary, AB, Canada, T2N 4N1

Running title: *P. zopfii* induces apoptosis in mastitis

*Corresponding author: Dr. Bo Han

hanbo@cau.edu.cn

Supplementary Figure 1. Cytochrome b (cytb) PCR and PCR-RFLP for identification of the *Prototheca zopfii* GT-II. The *Prototheca spp.* isolate recovered from the milk of a cow with clinical mastitis and used in our murine mastitis model was genotyped by cytb PCR and PCR-RFLP in comparison with a *P. zopfii* GT-I reference strain. **(A)** Cytb gene PCR results depicting a 644 bp PCR product for *P. zopfii* GT-I strain (Lane 1) that isolated from enviromnt and the *P. zopfii* GT-II isolated from the milk of a cow with clinical mastitis (Lane 2). DNA ladder: molecular-weight size marker. **(B)** Cytb PCR-RFLP results showing *P. zopfii* GT-I strain given 300 and 350 bp DNA fragments (from the 644 bp PCR product) after TaiI digestion (Lane 1) and the *P. zopfii* GT-II isolated from the milk of a cow with clinical mastitis given DNA fragments of 200 and 450 bp (from the 644 bp PCR product) after digestion with TaiI , compatible with *P. zopfii* GT-II (Lane 2). DNA ladder: molecular-weight size marker.

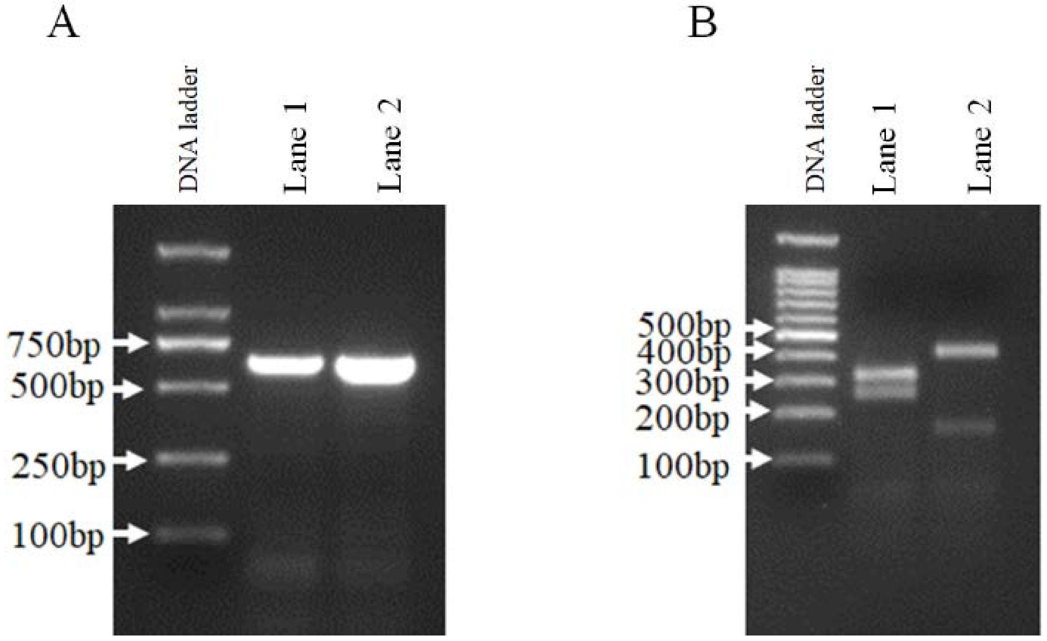

Supplement: Supplementary file 1 — Supplementary Figure 1. [file 41598_2020_57645_MOESM1_ESM.pdf]
