# Supplementary material for: Prototheca zopfii genotype II induces mitochondrial apoptosis in models of bovine mastitis
Source: Sci Rep. 2020 Jan 20;10:698. doi: 10.1038/s41598-020-57645-z (PMC6971270; doi:10.1038/s41598-020-57645-z)

Original articles for submission to *Scientific Reports*-

Prototheca zopfii genotype II induces mitochondrial apoptosis in models of bovine mastitis

Muhammad Shahid[1], Eduardo R. Cobo[2], Liben Chen[3], Paloma A. Cavalcante[2], Herman W. Barkema[2], Jian Gao[1], Siyu Xu[1], Yang Liu[1], Cameron G. Knight[4], John P. Kastelic[2], Bo Han[1]*

[1]Department of Clinical Veterinary Medicine, College of Veterinary Medicine, China Agricultural University, Beijing 100193, P.R. China

[2]Department of Production Animal Health, Faculty of Veterinary Medicine, University of Calgary, Calgary, AB, Canada, T2N 4N1

[3]Whiting School of Engineering, Johns Hopkins University, Baltimore, MD 21218, USA

[4]Department of Veterinary Clinical and Diagnositic Sciences, Faculty of Veterinary Medicine, University of Calgary, Calgary, AB, Canada, T2N 4N1

Running title: *P. zopfii* induces apoptosis in mastitis

*Corresponding author: Dr. Bo Han

hanbo@cau.edu.cn

Supplementary Figure 2. Transcriptomic expression of Bax and Apaf-1. mRNA expression of Bax and Apaf-1 after infection of *Prototheca zopfii* genotype GT-II infection in mouse mammary tissues (A and B), murine MECs (C and D) and murine macrophages (E and F), whereas in bMECs (G and H) mRNA quantified after infection of *Prototheca zopfii* genotype (GT)-I and -II infection by qPCR and expressed as fold change relative to uninfected samples. *$P < 0.05$, **$P < 0.01$.

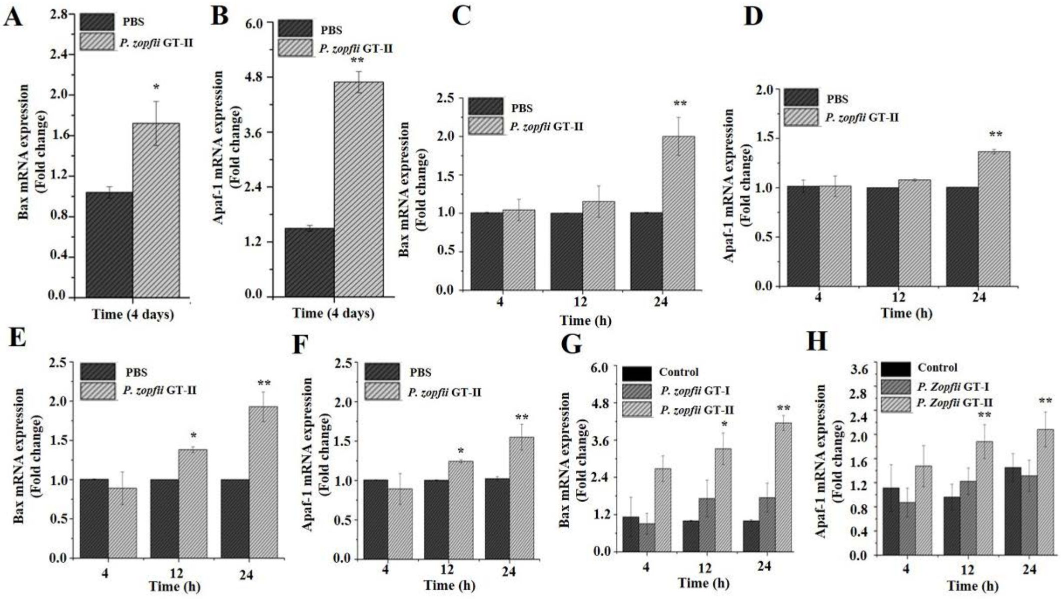

Supplement: Supplementary file 2 — Supplementary Figure 2. [file 41598_2020_57645_MOESM2_ESM.pdf]
